# Mitochondrial Copy Number and D-Loop Variants in Pompe Patients 

**DOI:** 10.22074/cellj.2016.4569

**Published:** 2016-08-24

**Authors:** Fatemeh Bahreini, Massoud Houshmand, Mohammad Hossein Modaresi, Hassan Tonekaboni, Shahriar Nafissi, Ferdoss Nazari, Seyed Mohammad Akrami

**Affiliations:** 1 Department of Medical Genetics, School of Medicine, Tehran University of Medical Sciences, Tehran, Iran; 2 Department of Medical Genetic, National Institute of Genetic Engineering and Biotechnology, Tehran, Iran; 3 Department of Pediatric Neurology, Shahid Beheshti University of Medical Sciences, Tehran, Iran; 4 Iranian Center for Neurological Research, Tehran University of Medical Sciences, Tehran, Iran

**Keywords:** Pompe, Mitochondrial DNA, D-Loop, Copy Number

## Abstract

**Objective:**

Pompe disease is a rare neuromuscular genetic disorder and is classified into two forms of early and late-onset. Over the past two decades, mitochondrial abnor- malities have been recognized as an important contributor to an array of neuromuscular diseases. We therefore aimed to compare mitochondrial copy number and mitochondrial displacement-loop sequence variation in infantile and adult Pompe patients.

**Materials and Methods:**

In this retrospective study, the mitochondrial D-loop sequence was analyzed by polymerase chain reaction (PCR) and direct sequencing to detect pos- sible variation in 28 Pompe patients (17 infants and 11 adults). Results were compared with 100 healthy controls and sequences of all individuals were compared with the Cam- bridge reference sequence. Real-time PCR was used to quantify mitochondrial DNA copy number.

**Results:**

Among 59 variants identified, 37(62.71%) were present in the infant group, 14(23.333%) in the adult group and 8(13.333%) in both groups. Mitochondrial copy number in infant patients was lower than adults (P<0.05). A significant frequency differ- ence was seen between the two groups for 12 single nucleotide polymorphism (SNP). A novel insertion (317-318 ins CCC) was observed in patients and six SNPs were iden- tified as neutral variants in controls. There was an inverse association between mito- chondrial copy number and D-loop variant number (r=0.54).

**Conclusion:**

The 317-318 ins CCC was detected as a new mitochondrial variant in Pompe patients.

## Introduction

Pompe disease (PD) or glycogen storage dis- ease type II is a rare neuromuscular genetic disor- der (1). Patients have a deficiency or lack of acid alpha-glucosidase (*GAA*) or acid maltase lysosomal enzyme due genetic mutations. It has an autosomal recessive pattern of inheritance (2) with an incidence ranging from 1:33,000 to 1:300,000 in different ethnicities (3). PD is classified by age of onset into two forms of infantile and late-onset (4). Accumulation of glycogen in vital organs such as cardiac, smooth and skeletal muscles leads to a broad spectrum of clinical features (5). The infantile form strongly affects cardiac, respiratory and skeletal muscles and enzyme activity is less than 1% of normal controls. They present hypertrophic cardiomyopathy, respiratory insufficiency, hypotonia and failure to thrive within the first few months of life. Late-onset Pompe is characterized by enzyme activity of about 2 to 40% and a prominent proximal skeletal involvement (4, 6).

Clinical manifestations are different according to disease severity (7, 8). Several mutations are recognized in the *GAA* gene, but the same mutation in two patients may lead to different manifestations and thus an alternative process seems to affect *GAA* mutations (9). Disease features are probably modified by unknown genetic and environmental factors (10).

Mitochondria generate adenosine thriphosphate (ATP) in cells. There is strong evidence that mitochondrial DNA (mtDNA) variation can enhance oxidative stress. It might also play a secondary role in additive damages (11). Over the past two decades, mitochondrial abnormalities have been recognized as important contributors to an array of neuromuscular diseases (12). Therefore mtDNA variation might be a modifying genetic factor that interacts with GAA mutation and may explain lack of strong genotype-phenotype correlation between *GAA* and PD (13). Knowledge about the role of mitochondria in the pathophysiology of PD is limited. Previous studies have shown abnormal mitochondria in Pompe patients, but were not focused on mitochondrial genes (14-17). These studies mentioned mitochondrial structural abnormalities as secondary rather than primary importance. Clinical phenotypes caused by mtDNA mutations are variable. Many phenotypes induced by mitochondrial diseases (cardiomyopathy, hypotonia, developmental delay and skeletal muscle manifestations) are the same as PD. The latter is the most common manifestation of mitochondrial disease. In addition, similar to PD, infantile mitochondrial disease is typically more severe than its adult-on- set form. Over the last decade, most of the research on PD has been focused on treatment in spite of an unclear pathophysiology. Understanding the pathogenicity of PD is necessary to therapy. For example, cardiac muscle responds well to therapy in contrast to skeletal muscle. Also, it is not clear why it is such a difficult target for enzyme replacement therapy (ERT). Poor response of skeletal muscle to therapy led us to examine other probable intervening genetics factors.

Human mtDNA comprises a 16569-base pair double strand circular genome, which encodes 13 proteins (from 37 genes) of the respiratory chain, 2 rRNAs and 22 tRNAs (18, 19). D-loop is the regulatory part of mtDNA, which has major role in transcription and replication, and is 1122 bp long (20). D-loop contains two hyper-variable regions (HVR1 at nucleotides 16024-16383 and HVR2 at nucleotides 57-372) (21, 22). There is also a tandem repeat of poly C in D-loop region from 303 to 315 nucleotides. Since D-loop has a regulatory role in mtDNA replication and transcription, mutations in this region might have a significant effect on copy number and gene expression of the mitochondrial genome, thus potentially disturbing mitochondrial function, oxidative phosphorylation (OXPHOS) and ATP production.

If mitochondrial dysfunction contributes to pathogenesis, ameliorating its effects could modify clinical symptoms of patients. In addition, identification of mitochondrial mutations or polymorphisms specific to infantile or adult forms of the disease may be useful as possible biomarkers. Therefore, this study investigated mitochondrial copy number and mitochondrial D-loop region variation in infantile and adult Pompe patients of Iranian origin.

## Materials and Methods

### Subjects

In this retrospective study, we recruited 28 Pompe patients (15 male and 13 female) from the Department of Neurology of Shariati and Mofid hospitals between December 2013 and February 2015. There were 17 and 11 infant and adult patients, respectively. Moreover, 100 healthy controls were recruited (17 infants and 83 adults). An informed consent was obtained from each participant or parents in the case of infant cases. PD was diagnosed in participants based on clinical findings by two expert neurologists, measurement of *GAA* biochemical activity or mutation detection in the *GAA*. Patients with no family history of mitochondrial or major neuromuscular disorders such as Duchene muscular dystrophy were included. The study was approved by the Ethical Committee of Tehran University of Medical Sciences.

### DNA extraction

DNA was extracted from whole blood by standard salting out protocol and using QIAamp DNA Blood Mini Kit according to the manufacturer’s instructions (QIAGEN, Germany). Quantity and quality of DNA were checked by NanoDrop ND- 1000 (NanoDrop Technologies, USA) at 260/280 nm and running on agarose gel (1%), respectively.

### Polymerase chain reaction-sequencing analysis 

Polymerase chain reaction (PCR) was performed with two pairs of primers

F: 5ˊ-GATCACAGGTCTATCACCCT-3ˊ

R: 5ˊ-AGTACACTTACCATGTTACG-3´ and

F: 5ˊ-CTCCTGCTTGCAACTATAGC-3ˊ

R: 5ˊ-GCTCCGGCTCCAGCGTCTGC-3ˊ

to amplify the entire D-loop as described previously (23). PCR master mix included 5 ng of genomic DNA, 0.8 μL of each primer (10 pmol), 0.2 mM of each deoxynucleoside triphosphate (dNTP), 1.5 mM MgCl_2_ and 1 U of Taq polymerase enzyme (CinnaGen, Inc, Iran). PCR cycling conditions for the first pair of primers were an initial denaturation at 94˚C for 4 minutes, followed by 35 cycles of denaturation at 94˚C for 60 seconds, annealing at 57˚C for 35 seconds and extension at 72˚C for 35 seconds, and a final extension at 72˚ C for 7 minutes. PCR conditions for the second pair of primers were an initial denaturation at 95˚C for 5 minutes, followed by 35 cycles of denaturation at 94˚C for 60 seconds, annealing at 58˚C for 60 seconds and extension at 72˚C for 35 seconds, and a final extension at 72˚C for 5 minutes. PCR-amplified fragments were sequenced by Macrogen (South Korea) using the same PCR primers in two directions and series of overlapping primers to cover all regions of interest for more accurate results. Finch TV 1.4 software (Geospiza, Inc., USA) was used to analyze sequences and were then checked using BLAST. The results were compared with the Cambridge reference sequence (20).

### Determination of mitochondrial DNA copy number

The mtDNA copy number was determined using a real-time PCR assay. In brief, to quantify mtDNA content relative to nuclear DNA (nDNA), primers for specific amplification of ND2 of mtDNA and nDNA-encoded β-actin gene were selected as described previously (24).

Forward and reverse primers as follow:

ND2: F: 5ˊ-CCCTTACCACGCTACTCCTA-3ˊ
  R: 5ˊ-GGCGGGAGAAGTAGATTGAA-3ˊ (279 bp);
β-actin: F: 5ˊ-ATCATGTTTGAGACCTTCAACA- 3ˊ
  R: 5´-CATCTCTTGCTCGAAATCCA-3ˊ (318 bp).

Real-time PCR was performed on a Corbett 6000 PCR-Real-time Detection System with a total volume of 20 μL reaction mixture containing 1 μL DNA template (5 ng), 10 μL SYBR Green PCR Master Mix (Takara, Japan), 8 μL nuclease- free water and 0.5 μL of each primer (10 pmol). Real time PCR protocol was an initial activation step at 95˚C for 10 seconds followed by 40 cycles including a denaturation step at 95˚C for 12 seconds and an annealing step at 60˚C for 35 seconds. Melting curve analysis was used to validate a PCR product for each primer pair. The copy number of mtDNA D-loop region in each tested specimen was then normalized against that of β-actin to calculate relative mtDNA copy number based on the 2^-ΔΔCt^ relative expression formula. Each measurement was repeated in duplicate and a nontemplate control was included in each experiment

### Statistical methods

Participant characteristics, mitochondrial D-loop variants and copy number were described as mean ± SD by SPSS statistical software for windows (IBM version 16, USA). Fisher’s exact and Chi- square tests were used to compare frequency of mitochondrial D-loop variants in infantile and adult Pompe patients, while a linear regression model was used to determine correlation between mtDNA copy number and D-loop variants numbers. P<0.05 was considered statistically significant.

### Results

**Fig.1:**
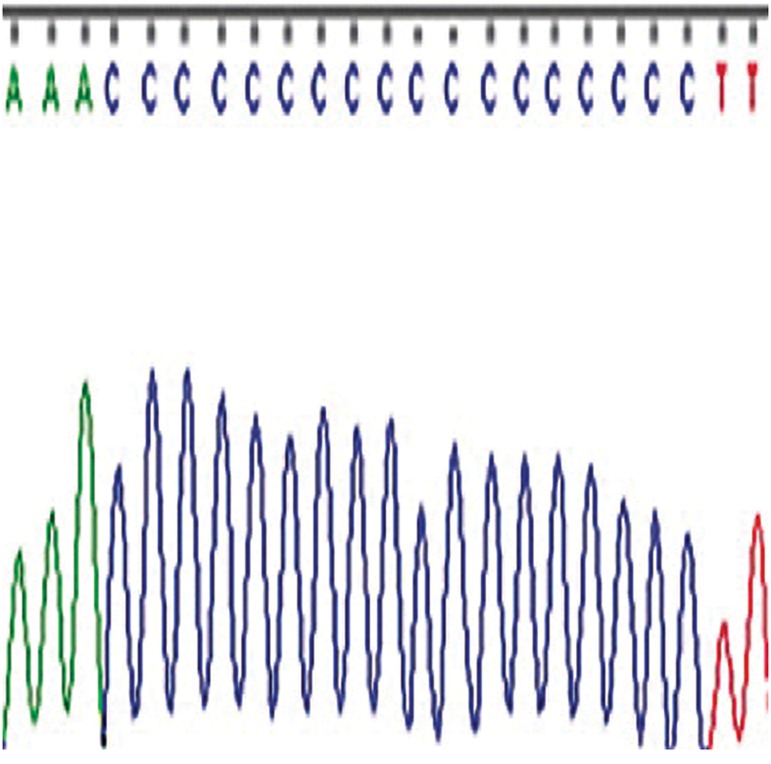
Electropherogram of the 317-318 insertion (CCC) with homoplasy.

Patients’ characteristics are shown in Table 1. The mean age of the control group was 23.29 ± 3.12 years. Fisher’s exact test showed no statistically significant difference between infant and adult patient groups regarding sex distribution (P=0.687) and family history (P=0.417). Screening of D-loop region in 28 cases of Pompe patients resulted in the identification of 59 variants. The variants absent in MITOMAP (www. mitomap.org) and other databases were checked in controls. A significant frequency difference was seen between the two groups for 12 SNPs (P<0.05, Table 2). Six SNPs were found as normal variants in controls compared with patients (Table 2, T152C, 514- 515 del CA, A272G, T16352C, G16319A, A73G). From the variants seen in the patient group, 317-318 ins CCC was novel (Fig.1). 

**Table 1:** Baseline clinical characteristics of Pompe patients


Characteristics	Infant	Adult

Gender, (n)		
Male	10	5
Female	7	6
Age of onset (Y, mean ± SD)	0.62 ± 0.43	21.7 ± 12.1
Muscular Pain, (n)		
No	NA	9
Yes	NA	0
Creatine kinase (U/L, mean ± SD)	1316.58 ± 327.11	611.37 ± 211.62
Vital capacity (cm^3^ , mean ± SD)	NA	56.14 ± 28.08
Walton score (mean ± SD)	NA	2.71 ± 0.49
6 minute waking test (mean ± SD)	NA	369.43 ± 111.58
Family history, n (%)		
Yes	8 (47.06)	4 (37.36)
No	9 (52.94)	7 (63.63)


NA; Denotes values that were not measurable, SD; Denotes standard deviation, and n (%); Denotes number (per- cent) of patients in each group. Walton score range is 0 to 10, in which 0 and 10 indicate the normal and the worst status, respectively.

**Table 2:** Mitochondrial D-loop variants in infantile and adult Pompe patients


Infant/Adult	Nucleotide	R/NRa	Ho/Htb	Pompe +/-^c^	Control +/-^c^	P value

Adult	A62G	R	Ho	2 / 26	0 / 100	0.012
	A93G	R	Ho	2 / 26	0 / 100	0.012
	C194T	R	Ho	2 /26	0 / 100	0.012
	G207A	R	Ho	1 / 27	0 / 100	0.090
	A235G	R	Ho	1 / 27	1 / 99	0.425
	T310C	R	Ho	1 / 27	0 / 100	0.090
	310 D C7TC6	R	Ho	2 / 26	0 / 100	0.012
	310 D C8TC6	R	Ho	2 / 26	0 / 100	0.012
	317-318 ins CCC	NR	Ho	1 / 27	0 / 100	0.090
	A503G	R	Ho	1 / 27	2 / 98	0.737
	513 ins GCA	R	Ho	1 / 27	0 / 100	0.090
	G16222T	R	Ho	3 / 25	9 / 91	0.786
	C16261T	R	Ho	1 / 27	10 / 90	0.255
	C16266T	R	Ho	2 / 26	0 / 100	0.012
Infant	G103A	R	He	1 / 27	0 / 100	0.090
	C150T	R	Ho	1 / 27	0 / 100	0.090
	C151T	R	Ho	2 / 26	8 / 92	0.882
	T152C	R	Ho	4 / 24	50 / 50	0.002
	A153G	R	Ho	1 / 27	16 / 84	0.052
	G185A	R	Ho	1 / 27	0 / 100	0.090
	G228A	R	Ho	1 / 27	0 / 100	0.090
	C242T	R	Ho	1 / 27	15 / 85	0.07
	A272G	R	Ho	1 / 27	100 / 0	<0.001
	C295T	R	Ho	3 / 25	12 / 88	0.853
	C456T	R	Ho	1 / 27	0 / 100	0.090
	C462T	R	Ho	3 / 25	0 / 100	0.003
	T480C	R	Ho	1 / 27	0 / 100	0.090
	T489C	R	Ho	6 / 22	0 / 100	<0.001
	514-515 del CA	R	Ho	3 / 25	33 / 67	0.028
	C553A	R	Ho	1 / 27	1 / 99	0.425
	G709A	R	Ho	1 / 27	0 / 100	0.090
	A16051G	R	Ho	2 /26	4 / 96	0.493
	G16145A	R	Ho	1/ 27	7 / 93	0.451
	A16163G	R	Ho	1 / 27	4 / 93	0.822
	A16183C	R	Ho	1 / 27	8 / 92	0.372
	T16189C	R	Ho	1 / 27	4 / 96	0.821
	C16248T	R	Ho	1 / 27	1 / 99	0.425
	C16256T	R	Ho	1 / 27	4 / p6	0.493
	C16278T	R	Ho	1 / 27	7 / 93	0.450
	C16294T	R	Ho	1 / 27	1 / 99	0.425
	C16295T	R	Ho	2 / 26	1 / 99	0.068
	T16298C	R	Ho	1 / 27	1 / 99	0.425
	A16300G	R	Ho	1 / 27	1 / 99	0.425
	T16304C	R	Ho	2 / 26	3 / 97	0.326
	A16309G	R	Ho	1 / 27	10 / 90	0.254
	A16318T	R	Ho	1 / 27	0 / 100	0.090
	C16320T	R	Ho	1 / 27	2 / 98	0.737
	T16352C	R	Ho	1 / 27	100 / 0	<0.001
	C16354T	R	Ho	1 / 27	0 / 100	0.090
	T16362C	R	Ho	2 / 26	1 / 99	0.068
	G16390A	R	Ho	2 / 26	7 / 93	0.979
Both	T72C	R	Ho	2 / 26	0 / 100	0.012
	A73G	R	Ho	10 / 18	81 / 19	<0.001
	T146C	R	Ho	6 / 22	0 /100	<0.001
	T195C	R	Ho	7 / 21	2 / 98	<0.001
	A211G	R	Ho	1 / 27	0 / 100	0.090
	A263G	R	Ho	23 / 4	70 / 30	0.214
	A385G	R	Ho	2 / 26	19 / 81	0.146
	T16311C	R	Ho	2 / 26	2 / 98	0.178


^a^, R, NR; Denote reported and non-reported variants respectively in previous studies, ^b^, Ho, Ht; Denote homoplasmy and heteroplasmy, respectively, and ^c^, + and -; Show positive and negative results for each variant, respectively

**Fig.2:**
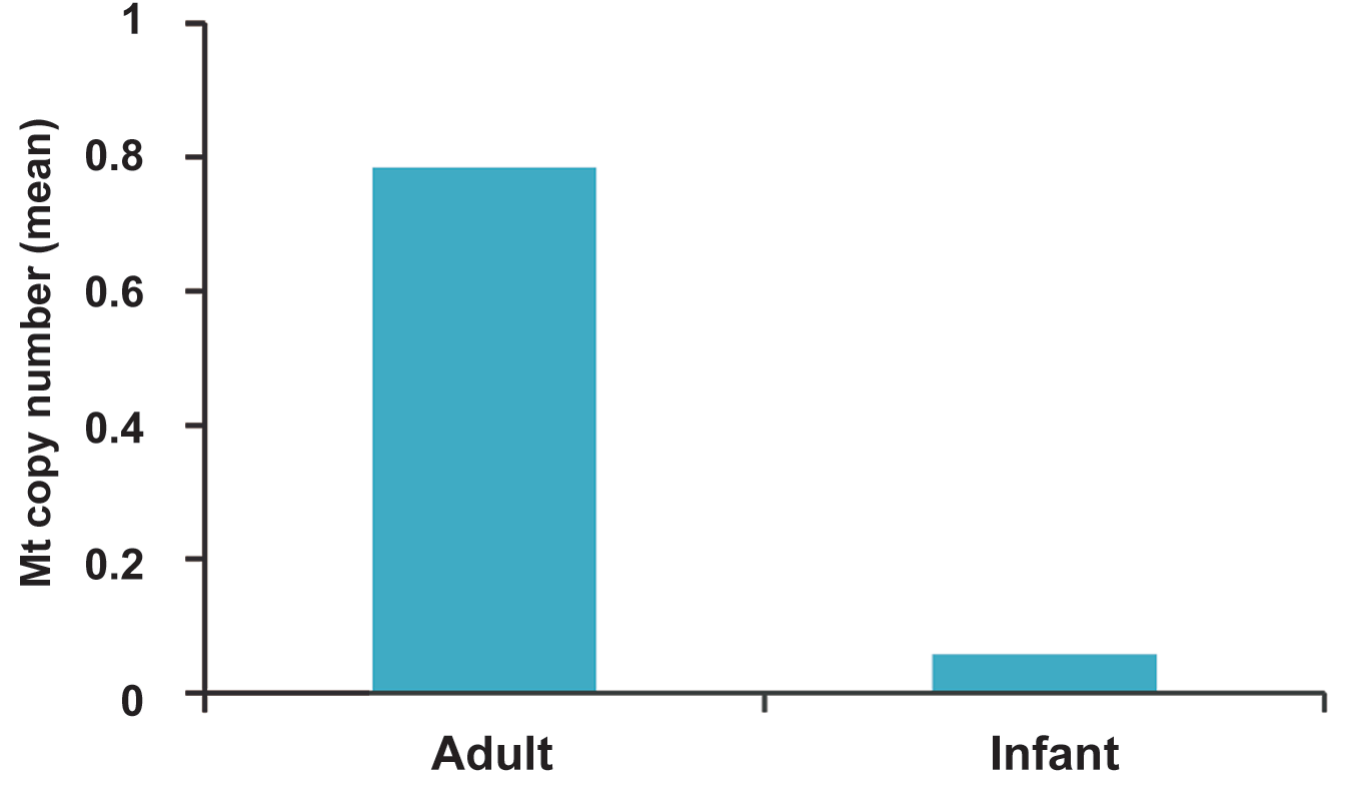
Mitochondrial copy number ratio in infantile and adult Pompe patients versus controls.

Of the 59 variants, 37 (62.71%) variants were observed in the infant group, 14 (23.333%) in the adult group and 8 (13.333%) in both groups. The mean number of D-loop region variation in each patient was 6.0714. The mean number of variants in infants and adults were 7.058 and 4.54, respectively. All variants were homoplas- mic except G103A. Mitochondrial copy number in infantile patients was lower than adult pa- tients (P<0.001, Fig.2), while there was no sig- nificant difference between infantile and adult controls (P=0.12).

**Fig.3:**
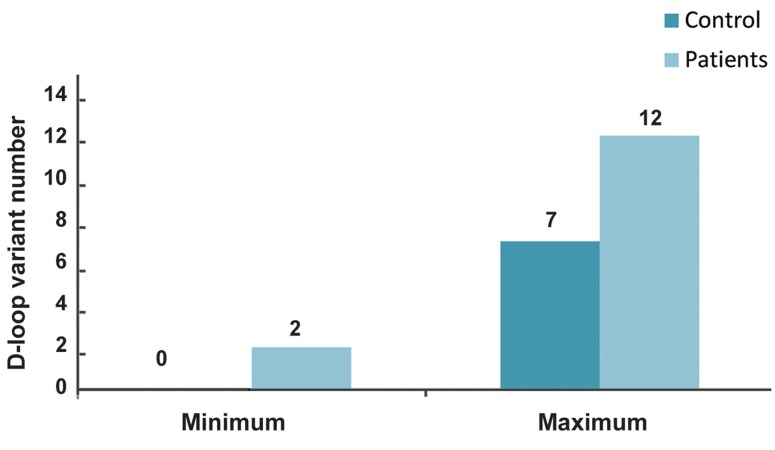
Minimum and maximum D-loop variant number in Pompe patients and controls.

The range of D-loop variant count among all patients was 2-12 while this was 0-7 in the controls (Fig.3). This study showed that D-loop variant number in infantile patients is higher than adult patients. Chi-square test showed a meaningful difference in the distribution of D- loop variant counts between the infant and adult groups (P=0.038). Some infant patients had 10 variants or more in the D-loop region, while adults had nine variants or less. However, D- loop variant count in healthy adults was more than in healthy infants (P=0.041).

**Fig.4:**
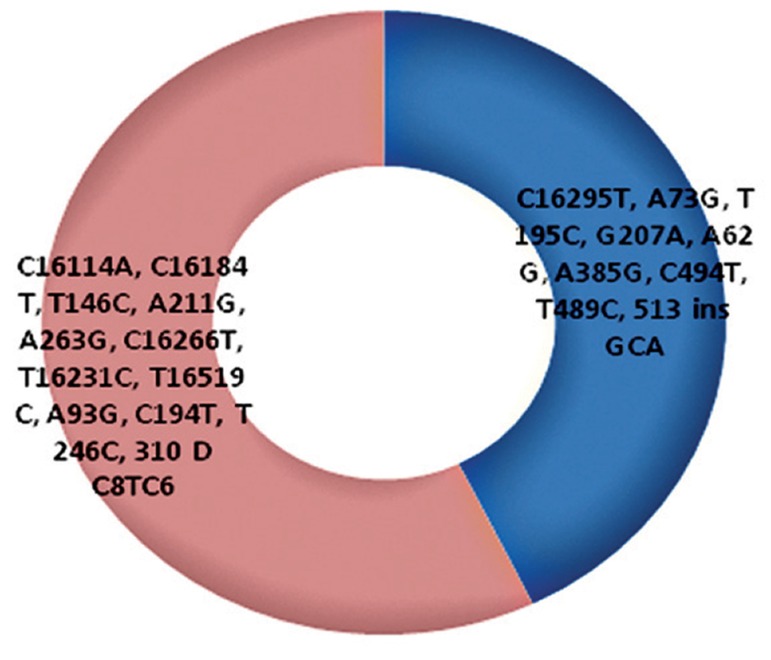
Common/unique variants among four affected sib pairs. Blue area shows common variants and pink area shows unique variants in the sib pair patients.

Among the patients in this study, four affected sib pairs were present. They were compared based on their variants. 57.14% of all variants (21 vari- ants) were common in sib pairs (Fig.4).

**Fig.5:**
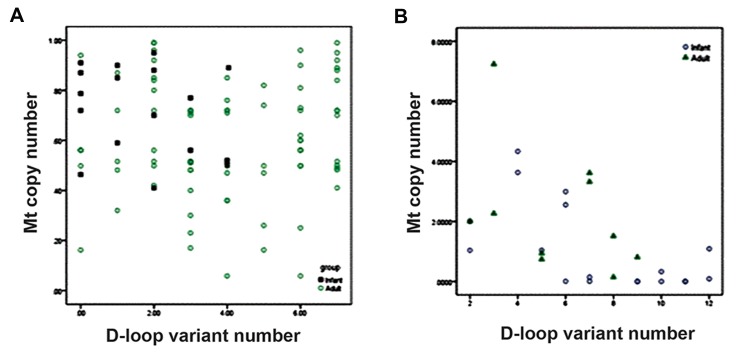
A. Scatter plot of mtDNA copy number and D-loop variant count in controls and B. Scatter plot between mtDNA copy number and D-loop variant count in patients. Correlation coefficient R and linear regression line of best of fit should be add.

Linear regression analysis (Fig.5) showed a statisti- cally significant inverse correlation (P=0.003, correla- tion coefficient R=0.54) between D-loop variant count and mtDNA copy number in patients (Fig.5B). There was no correlation between D-loop variant number and mtDNA copy number in controls (Fig.5A).

## Discussion

We evaluated mitochondrial copy number and mitochondrial D-loop variants in infantile and adult Pompe patients to investigate the potential role of mitochondria in PD. There are common clinical manifestations (variable expression and variable age of onset) between Pompe and mito- chondrial disorders. Some studies have indicated that phenotypic expression of PD has significant variability in affected individuals with identical causal mutations (22).

As mitochondria are controlled by nuclear and mitochondrial genes, mutations in both set of genes may affect mitochondrial function and in- crease oxidative stress. On the other hand, increas- ing oxidative stress is a secondary factor that re- sults in mitochondrial dysfunction.

Engel and Dale (25) showed a large number of abnormal mitochondria in skeletal muscle biopsy derived from a patient with adult onset disease. Paracrystalline inclusions were reported in numer- ous mitochondria among adult patients (15, 26). Hudgson (27) revealed that large subsarcolemmal mitochondrial aggregates exist in an adult patient. Furthermore, Verity reported enlarged “pleomor- phic” mitochondria with distorted cristae in mus- cular tissue of an infant (16). Recently, Huang ob- served dysfunctional mitochondria with swollen cristae in induced pluripotent stem cells (iPSCs) derived from fibroblasts of two patients with PD and showed that mitochondrial dysfunction is one of the pathophysiological features of PD cells (10, 27). These studies reported abnormalities in mito- chondrial structure, but we evaluated mtDNA in Pompe patients.

D-loop part is a major region that acts as the ori- gin of replication of mtDNA and contains essential elements for transcription and replication of mito- chondrial genes. D-loop has been shown to be a mutation ‘hot spot’ in some disorders and is more vulnerable to numerous damages. The rate of mu- tation in D-loop is much higher than other parts of mtDNA and such alterations perhaps interfere with the sequence of promoters and modify the af- finity of binding to the inducer or modulators that is part of the transcription machine (28). In the pre- sent study, a large number of point mutations were observed in the D-loop region of mtDNA. Several variants were within nucleotides 110-520. These variants include 146, 150, 152, 153, 203, 228, 263, 295, 456, 489 and 513. These variants were also in cardiomyopathy cases (29). It may thus interfere with mitochondrial replication, transcription and mtDNA copy number. In spite of decreasing mtD- NA copy number in the infantile group, D-loop variant count increased. The infantile mean variant count was more than that of the adult group.

We report in this study that almost all D-loop variation in Pompe patients were homoplasmic. The D310 mutations were seen in 17.857% of patients. The D310 region has been recently rec- ognized as a frequent hot spot of insertion muta- tions in some disorders (30, 31). This polymor- phic c-stretch (CCCCCCCTCCCCC) has a role in the formation of persistent RNA-DNA hybrid, which is essential for mtDNA heavy strand rep- lication (31). The T152C variant was also seen in child respiratory morbidity (32) and reported to be present in H, U and K haplogroups. The role of haplogroups is emphasized for several disorders like age-related macular degeneration (33). This variation is associated with haplogroup H and can increase the risk of Parkinson disease (34), while in this study the variant was not significantly over- represented in the case group. T489C and T146C were observed frequently in the study, both being more frequent in some populations and disorders (35, 36). The T146 variant has been detected in mi- tochondrial myopathies, hearing loss, mitochon- drial encephalomyopathy and ovarian cancer (37, 38). T195C and C462T variants have been associ- ated with repeated pregnancy loss and psychiatric disorders (22). The A73G variant has been report- ed in Alzheimer’s, hearing loss, hypertrophic car- diomyopathy and infantile cardiomyopathy. The A263G variant has been associated with muscle pathology, auditory neuropathy, hypertrophic car- diomyopathy, idiopathic cardiopathy and infantile cardiomyopathy (39). The C16278T has been seen in hearing loss, idiopathic cardiomyopathy, Leigh syndrome, mitochondrial encephalomyopathy and mitochondrial myopathy. These D-loop variants due to mimic Pompe clinical presentations (40).

The T16189C variant has been reported in car- diomyopathy and diabetes and has been suggested to affect mitochondrial DNA replication (39, 41). These variants may change the affinity of transcrip- tion factors and other cis acting elements in the D- loop region and affect mitochondrial function. The role of mitochondrial variants in PD is unknown. This study evaluated mtDNA copy number and the D-loop region variants for the first time in infan- tile and adult Pompe patients of Iranian origin. It seems that there is a negative correlation between mtDNA variation and mtDNA copy number when analyzing severe PD patients. Other mitochondrial variants within the same haplogroup may have an effect on severity of the disease and may act in synergy with *GAA* mutations.

## Conclusion

This is the first study regarding mitochondrial evaluation of infantile and adult Pompe patients of Iranian origin. We identified a novel variant (317-318 ins CCC) in Pompe patients. There was an inverse correlation between mean D-loop variant count and mtDNA copy number. A significant frequency difference was seen between the two groups for nearly a fifth of SNPs. 

MtDNA copy number and variant count were different between adult versus infant patients. These differences were large in infants and these results are coordinated with phenotype intensity. It seems that mitochondrial variants may have a secondary role in the pathogenesis of PD. Understanding the role of mitochondria in the pathogenesis of PD could pave the way for the development of new therapeutic strategies. 
